# Lived experience of avoidable healthcare harm in prisons in England: a qualitative study

**DOI:** 10.1186/s40352-026-00417-0

**Published:** 2026-07-23

**Authors:** Mel Jordan, Sandra Flynn, Ellie Thompson, Deborah Buck, Claire Muller, Noor Butt, Basharat Hussain, Jenny Shaw, Darren M. Ashcroft, Antony Avery, Andrew Carson-Stevens, Richard N Keers, Isobel Joy McFadzean, Tim Millar, Maria Panagioti, Verity Wainwright

**Affiliations:** 1 School of Sociology and Social Policy, Law and Social Sciences, University of Nottingham, Nottingham, UK; 2 Division of Psychology and Mental Health, School of Health Sciences, Faculty of Biology, Medicine and Health, University of Manchester, Manchester, UK; 3 School of Medicine, University of Nottingham, Nottingham, UK; 4 Division of Pharmacy and Optometry, School of Health Sciences, Faculty of Biology, Medicine and Health, University of Manchester, Manchester, UK; 5 National Institute for Health and Care Research (NIHR) Greater Manchester Patient Safety Research Collaboration (GM PSRC), Manchester, UK; 6 Division of Population Medicine, School of Medicine, Cardiff University, Cardiff, UK; 7 Division of Population Health, Health Services Research and Primary Care, School of Health Sciences, Faculty of Biology, Medicine and Health, University of Manchester, Manchester, UK

## Abstract

**Background:**

People in prison experience high rates of physical and mental health problems and although prison provides opportunities to improve health and wellbeing, they often experience worse health outcomes. This study aimed to explore the perspectives of staff and people in prison, considering their experiences of perceived avoidable healthcare-associated harm and the factors they felt influenced prevention of harm.

**Methods:**

Qualitative, semi-structured, interviews were conducted with 32 prison staff and commissioners and 23 people in prison across five prisons in England. Interviews were conducted between August 2022 and January 2024 and were analysed thematically.

**Results:**

Problems with access to timely and appropriate healthcare were commonly raised by participants in relation to issues around medication management and access to healthcare. Prison policies and practice were highlighted by participants as factors that influenced the prevention of harm; interviewees referred to the impact of staffing capacity and the skillsets of staff in terms of trauma and mental health, the impact this had on relationships between staff and people in prison, as well as challenges related to communication and coordination between teams within the prison.

**Conclusion:**

Experiences of perceived avoidable healthcare-associated harm in prisons centred on medication management issues and difficulties with accessing healthcare. Recruiting and retaining skilled staff and systems lacking communication and coordination were considered important factors influential to harm prevention. A systems-based approach is needed to improve patient safety considering how integration and communication can be improved but this is needed alongside improved health literacy and interventions to increase autonomy that are led by people in prison.

## Background

People who reside in prison are vulnerable; they experience high rates of mental and physical health needs; acute and long-term conditions and substance use problems (Baranyi et al., 2022; Fazel et al., 2016; Peters et al., 2015; Semenza & Grosholz, 2019; World Health Organization [WHO], 2014). Residing within prison provides an opportunity for healthcare engagement, assessment, diagnosis, and treatment (Fazel & Baillargeon, 2011). However, the delivery of healthcare services in prison is complex, with secure environments creating specific organisational and delivery challenges (McFadzean et al., 2023, 2025; Nuffield Trust, 2021). People in prison have limited opportunities to make choices about their care (Keers et al., 2023) and overcrowding, staffing, and resource problems further complicate healthcare delivery (Prison Reform Trust, 2025).

In England and Wales, prison healthcare is based on the principle of equivalence (Royal College of General Practitioners, 2018), however, people in prison access healthcare services three times more frequently than people in the community and have worse health outcomes (Marshall et al., 2001). Although not a new concern (Fazel & Baillargeon, 2011), healthcare providers are facing pressure to improve patient safety in prisons (McFadzean et al., 2023, 2025; Iacobucci, 2024; Ismail, 2022). A recent review of prison deaths identified that people in prison die around 20 years younger than their counterparts in the community (National Confidential Enquiry into Patient Outcome and Death, 2024). Eradicating all harms may not be possible but better understanding what harm is preventable will help guide healthcare provision in prison and ultimately reduce health inequalities (Kinner & Young, 2018).

In primary care in England, the incidence of significant harm estimated to be least ‘probably’ avoidable is 35.6 per 100,000 patient years; the main sources of harm were diagnostic error, medication incidents and delayed referrals (Avery et al., 2021). However, we know less about avoidable harm in prison healthcare. Avoidable healthcare-associated harm in prisons has been defined as an incident that could have probably or totally been avoided, using a two-tier judgement considering how community care would be assessed, and additionally considering the restrictions of the prison regime (Keers et al., 2023). Such a definition allows the assessment of what avoidable healthcare harms occur and their prevalence (Carson-Stevens et al., 2024). However, it is essential to complement this by exploring the perspectives of staff and people in prison to illuminate how they interpret and experience avoidable healthcare harm (Panagioti et al., 2019; Vincent et al., 2014); this has the potential to inform policy and practice improvements aimed at reducing harm in prison. This paper presents qualitative work conducted as part of a wider programme exploring avoidable harm in prison healthcare (Keers et al., 2023; Carson-Stevens et al., 2024) to understand how harm is experienced by those working and living in that environment.

## Methods

### Design

The study utilised qualitative semi-structured interviews to collect data that explored experiences of avoidable healthcare-associated harm in prisons in England from the perspectives of staff and people in prison. A qualitative study was deemed most appropriate as it enabled the research team to gather deeper insights into these experiences than a quantitative approach would allow (Lim, 2025).

### Setting and participant selection

Five prisons in the Northwest and Yorkshire and Humber regions of England were purposively sampled to take part. Prisons were selected pragmatically to ensure the different security categories (i.e., A, B, C, and D) and a women’s prison were represented.

Staff and people in prison were purposely sampled. Staff included recruitment of commissioners (i.e., those with prison oversight in health and justice roles) and people in various roles at the participating sites (i.e., including prison residential and healthcare staff). People in prison were eligible to participate if they self-reported having accessed any healthcare while in prison; we did not target recruitment to people who reported experiencing avoidable harm. A member of staff was established as a link person at each prison to facilitate recruitment and circulate study information to potential participants. Information was circulated via staff lists and the research team also utilised professional contacts to invite commissioners to take part. To recruit people in prison, posters were displayed in healthcare areas and the link person also circulated study information directly. Where possible, researchers were also present to provide further information to anyone who expressed interest in taking part.

### Data collection

An interview guide was used to explore participants’ experience of avoidable healthcare-associated harm and factors they felt influenced the prevention of harm. The same overall interview guide was used for both staff participants and people in prison with specific prompts used accordingly. Five short vignettes (i.e., two or three sentences) outlining hypothetical scenarios of avoidable harm were also developed to help facilitate discussion with participants and their understanding of avoidable harm. The vignettes were used to help ground discussions and provide conceptual clarity. Both the interview guide and vignettes were developed by the research team using insights gathered from a previous phase of the study (Keers et al., 2023; McFadzean et al., 2023) to ensure conceptual coherence, whilst recognising that participants’ responses would be shaped by their own interpretations and experiences. The vignettes were provided at the start of the interview to allow reflection throughout the interview.

All participants took part in one individual interview, facilitated by one of two Research Assistants (ET and CM). No one else was present for the interviews. Interviews were predominantly conducted in person, although some staff interviews were conducted remotely to support flexibility. All interviews were audio recorded and transcribed by the research team. Interviews lasted on average 45 min and data collection took place between August 2022 and January 2024.

### Data analysis

Interviews were transcribed verbatim and checked by a researcher for confidentiality and accuracy; this involved listening to the audio recording and checking this against the transcript and anonymising any information that had the potential to render an individual identifiable. Interviews were analysed thematically by a team of five researchers (MJ, DB, NB, BH and ET) to move from raw data to organised data, coding, themes, findings and analysis. Transcripts were read and re-read to achieve familiarity with the content of each interview. Data was then purposively extracted from the transcripts and organised and presented thematically (i.e., experiences of perceived avoidable harm and factors influencing the prevention of harm). Excel was used to manage this process with a datasheet created for each set of interviews (i.e., one for staff participants and one for people in prison); separate tabs were used to organise and differentiate coding by the analysts that captured harm events described by participants and contextual barriers to preventing harm. Each datasheet contained the transcript identifier; the extract from the interview; line number of the transcript; analyst name; and a descriptive finding (i.e., a summary of what the extract was about). The next step brought together descriptive findings with analytical concepts, with lengthier summary paragraphs written to explain the culminated data for that topic. The analysis focused on identifying shared patterns across the two datasets. Frequent meetings between the research team were held to review the analysis process and agree the emergent analysis. Reflexivity was supported by the team’s multidisciplinary backgrounds and informed by reflection on earlier insights gained (Keers et al., 2023; McFadzean et al., 2023), which helped balance interpretation through collaborative analysis.

### Ethical considerations

Ethical approval for the study was granted by NHS REC (Wales Research Ethics Committee 3; reference 19/WA/0291) and His Majesty’s Prison and Probation Service (HMPPS; reference 2019-322) and was conducted in accordance with the Declaration of Helsinki.

All participants were provided with detailed information about the study via a Participant Information Sheet and given time to consider whether they wanted to take part. All participants provided written informed consent and our role as researchers, with no influence over prison status, privileges or care, was communicated to all participants. Participation in the study was confidential, known only to the research team, and the limits of confidentiality were explained to all participants. A Data Management Plan was in place for the study to ensure data were handled securely and confidentially. A distress protocol was in place to support participants if needed, however this was not required. Any ongoing healthcare issues that were uncovered during the research were reported to the prison directly.

## Results

In total, 55 interviews were conducted. Details of participants’ characteristics are provided in Table 1.

**Table 1:** Participant characteristics

Category	Staff Participants	People in prison
No. of participants	32	23
Roles	Prison officer, prison governor, chaplain, custodial, safer custody manager, healthcare and mental health nurse, GP, pharmacist, healthcare manager, psychologist.	Not applicable
Duration of role	2 months–21 years	Not applicable
Gender	Male: 16 (50%), Female: 16 (50%)	Male: 19 (83%), Female: 3 (13%), Non-binary: 1 (4%)
Age range	24–59 years (M = 52)	19–62 years (M = 43)
Ethnicity	White British: 26 (81%)	White British: 17 (74%)
Sentenced	Not applicable	20 (87%) (sentence duration ranged from 3 years to Life/Imprisonment for Public Protection [IPP])
Health services accessed	Not applicable	Hospital services, GP appointments, mental health care, substance dependence services, dentists, physiotherapy, podiatry, pharmacy, and various healthcare clinics.

Access to timely and appropriate healthcare were problems experienced by staff and people in prison. Workforce capability and operational cohesion issues were identified as factors that influenced the prevention of harm. These findings are discussed with illustrative quotes.

### Problems with access to timely and appropriate healthcare

Staff and people in prison reported that poor access to healthcare was the most experienced avoidable healthcare-associated harm. Participants identified that medication management was a key area of concern and described how this impacted them. Equally, missing healthcare appointments, either because of prison security requirements, poor communication or lack of autonomy over self-care, were reported to lead to delays in care and treatment.

#### Issues with medications management

Inadequate medications management was identified as a recurring theme. Pharmacy dispensing and prescription incidents were commonly reported alongside people in prison experiencing enforced medication changes, resulting in frustration. Many participants spoke about the problems accessing essential, routine and repeat medications as well as being administered incorrect doses and the impact this had on them. A staff member stated: *“Sometimes there can be avoidable aspects in relation to the pharmacy and medication side of it… maybe not ordering or maybe having a delay in ordering… if there’s a delay on that*,* that can have a delay in care given.”* (Gst03, Administrator).

There were also reports of failures to provide follow-up medicine post treatment, including post- surgery. One participant observed *“… the aftercare that I’ve seen with other people… the hospital sends you all the way back with medication*,* you might not get that medication when you’re supposed to get it.”* (Mpr25, person in prison). One participant spoke about the stresses related to medication, especially at times when [they] were particularly vulnerable:

*“I’ve had quite a stressful time. A lot has happened… they gave security [my] medication and the doctor wouldn’t let me have [it]… I come back from the chemo*,* and you can imagine… It was bad. I was shaking and they wouldn’t give me my medication. The doctor said*,* no you can’t have it ‘cause you’ve had a triple bypass. I got my [partner] to ring [cancer centre].”* (Ipr04, person in prison).

This experience emphasises the helplessness and loss of autonomy experienced by this person who was reliant on others outside of the prison to advocate for their needs. Another person in prison explained how issues around medication resulted in feelings of despondency and not feeling cared for:

*“You’re just part of a system and a process. I am able to kind of rationalise that and deal with that. but [.] some people don’t [.] and they’re not getting this*,* they’re not getting that*,* it does spiral a lot of people into this no one cares about me.”* (MPr23, person in prison).

Another echoed how the general lack of autonomy when it came to healthcare made them feel anxious: *“You’ve no control over your own destiny basically. You can’t just nip down and say*,* can I see a nurse? Because you’re just not allowed… It makes me feel very*,* very anxious.”* (MPr24, person in prison).

It was also reported that delays caused by poor medication management resulted in self-harm and destructive behaviour. One member of staff commented: *“… some self-harmed because the medication had been stopped or they’ve got the wrong medication or the medication isn’t working and they feel that’s their only option is to self-harm … they want the help*,* but they weren’t getting it.”* (Ist25, Prison Officer). A person in prison further explained how poor communication further compounds the issue:

*“…they’ve gone to the meds hatch to get their medication … and they said ‘no you’ve been taken off it’ … then that person has to go through multiple weeks waiting to get back to the GP … if they’re struggling … they might smash the cell up or something like that*,* get in trouble for it*,* but at least they’re going to get their meds.”* (Fpr21, person in prison).

#### Access to healthcare and missing healthcare appointments

Delays in healthcare provision resulted from practices and policies within the prison as well as individual choices. Participants expressed frustration at being recorded as ‘Did Not Attend’ (DNA) to appointments which were not attended due to escort delays, with prison officers not unlocking or escorting people in time or staff shortages:

*“…I think I had buzzer on for forty minutes the other day. And then you get a black mark because you’ve not gone to a healthcare appointment. But you’ve been trying to …”* (Mpr24, person in prison).

Individual choices around self-care were highlighted as a factor in accessing healthcare. People in prison admitted that at times they prioritised their limited recreation time or other activities such as work or education. One participant spoke about how using free time for recreation can take precedence: *“…because of limited regimes and people not being able to come out*,* people are going to rather prioritise their social over their healthcare appointment*,* unfortunately.”* (Ipr16, person in prison).

Some appointments were also missed due to individuals feeling anxious, worried about stigma, or the need to maintain privacy, particularly if attending clinics for mental health or phlebotomy clinics, the latter being associated with infectious diseases. A member of staff cited an example of this: *“…the Hep. C patients*,* we have a blood clinic for them. But probably …they think that everybody might know that blood clinic might be for Hep. C so they might not want to go.”* (Mst31, Pharmacist). A person in prison echoed this sentiment, suggesting more privacy could be offered:

*“It’s not great that you come in and you’ve got to explain the problem in front of a room full of lads… maybe if there were sort of a dividing wall put up and you had a bit more privacy to speak to the receptionist*,* that might help.”* (FPr07, person in prison).

### Workforce capability and operational cohesion

Participants discussed policies and practices that they perceived contributed to avoidable harm. The most discussed areas of concern were in relation to the staffing capacity, the skillset of staff and communication and coordination between teams.

#### Staffing capacity and variable staff skillsets

The inherent restrictions of the prison environment, including prolonged time in cells and the lack of autonomy of people in prison were also spoken about by staff. They acknowledged staff shortages were a key factor that contributed to issues accessing healthcare. The prison environment was also widely recognised as a challenge to hiring and retaining staff, with prison jobs perceived as less appealing than community positions: *“Sometimes they can start and then they find that prison is not the environment for them because… it’s an added pressure along with their actual job itself*,* which is pressure at the best of times.”* (GSt03, Administrator).

Many participants considered a shortage of skilled workers, and dependence on inexperienced or agency personnel, particularly in healthcare, to impact on the support provided: *“They just seem to not really have great levels of staff to support the prisoners and also agency staff doesn’t help because they don’t know the prisoners*,* which makes it worse because they can sometimes misconstrue people’s behaviours.”* (Fst18, Hub Manager).

More specifically, participants referred to the skills of personnel, and insufficient training, particularly in mental health and trauma-informed care, which were believed to lead to mismanagement of requirements, heighten the risk of self-harm, and add to strained relationships between staff and people in prison. One member of staff reflected on this lack of understanding around mental health among prison officers:

*“It comes back to stigma towards mental health… I think a lot of officers maybe aren’t trauma informed … If prisoners are presenting as aggressive*,* I might be able to understand it as fear which has turned into hostile behaviour.”* (Ist16, Assistant Psychologist).

Residential staff also considered the value of being better trauma-informed; a member of safer custody staff who had received some training in this regard commented: *“Understanding previous trauma … a lot of staff haven’t gone through that*,* and they’ve not had that experience and so they don’t understand the benefit of it.”* (FSt20, Safer Custody Officer). A person in prison also commented: “*Not everyone has the right skills. Not everyone has the right mindset for this job.”* (IPr16, person in prison).

#### Communication and co-ordination between healthcare and security teams

Communication barriers were a commonly reported issue. Staff commented that there were confidentiality constraints, with officers not being informed about the health conditions of people on prison, which further complicated their ability to provide appropriate support. Poor communication was noted between teams; one member of staff summarised how this can impact the person:

*“I think where people are not talking to each other*,* that’s where I think things fall down a lot. That’s where really*,* we missed opportunities because if we’re all holding a bit of the jigsaw*,* but we never bring it together*,* then we don’t understand the full picture*,* and so therefore we approach it in different ways*,* almost in silos*,* and in doing so*,* we’re not really making the impact that we could. … the cost here is not just financial*,* potentially the cost is harm to the individual*,* and you know the ultimate cost is death of the individual.”* (Fst23, Chaplain).

A person in prison further commented: *“There just needs to be more talking between them [healthcare and prison staff]*,* more respect… they should be working together.”* (Fpr07, person in prison). People in prison reported feeling frustrated at a lack of shared decision-making and not being involved in or consulted about their treatment needs: *“… it’s not the same consultative approach that you’d get on the outside*,* it’s a lot more dictatorial.”* (Fpr21, person in prison).

Staff participants felt that poor communication between healthcare and prison officers, combined with negative attitudes of some staff towards people in prison, results in a culture of mistrust and blame. Overall, a lack of collaborative working between healthcare services, but also within the prison more widely, was considered problematic:

*“As soon as this sort of silo-thinking and silo-blame*,* then that’s when we increase the risk… from an operational and a strategic point of view…. as soon as there is this… expectation that it’s ‘the responsibility of this body’ or ‘the responsibility of that body’*,* then there are gaps. And where there are gaps*,* there are risks.”* (Cst10, Mental Health Team).

Poor coordination between healthcare staff and prison officers was noted by participants. Staff explained that there were sometimes missed opportunities to refer people in prison to mental health services because of the prison officers’ limited understanding of mental health and available services in the prison mean issues are not picked up upon as efficiently as they could be. Similarly, staff within healthcare felt there was a limited understanding of overall prison routines which resulted in challenges when coordinating appointments. This was part of a wider conflict between healthcare services and the prison regime where attending healthcare is reliant on officer assistance: *“We [nurses] have no way of knowing what times their associations are going to be because it changes on a day-to-day basis.”* (Ist15, Nurse).

## Discussion

Interviews with staff and people residing in prisons explored experiences of avoidable healthcare-associated harm and the factors perceived to influence prevention of harm. This included medication management, such as prescription access and medication errors and changes. These impacted on the behaviour of people in prison, in particular feelings of frustration, self-harm and destructive behaviour but also in them feeling helpless, reinforcing their lack of autonomy. Delays in access to healthcare and missed healthcare appointments were common and often related to staffing and logistical challenges (e.g., lack of staff for escorts to appointments and coordination between prison and healthcare staff), leaving people in prison feeling frustrated that they are blamed for not attending when the appointment is then labelled as a DNA. Such issues have previously been identified in an analysis of patient safety incidents (McFadzean et al., 2023).

However, important insights have been gained by exploring the perspectives of staff and people in prison. For example, individuals choosing to prioritise other activities over attending healthcare appointments and issues around stigma and privacy were also factors felt to impact healthcare access and attendance. Challenges with the prison environment in terms of staffing and skillset variability were also acknowledged. Recruiting and retaining skilled and experienced staff was considered problematic. Experience and understanding of mental health and trauma-informed care was noted as essential to improve relationships between staff and people in prison and was felt to be hindered by staffing challenges across the prison and reliance on agency staff in healthcare. This impacted on the challenges of collaboration between prison and healthcare staff with each side of prison not having enough understanding of the other, resulting in missed opportunities for referrals and difficulties scheduling appointments. Coordination between different areas of the prison was further hampered by poor communication between members of staff and between prison and healthcare teams, further negatively influencing healthcare support provided.

Medications management within prisons is a critical area for addressing avoidable healthcare harm (McFadzean et al., 2023, 2025). Gaps in medication from community care due to prescription ordering or availability issues is a concern in custodial settings (Wright et al., 2021), demonstrating the need for alternative medication access plans. To enhance continuity of care, structured handovers between pharmacy and healthcare should be introduced (Favril et al., 2024), and timely review with people in prison following a prescription change is essential. This should reduce medication incidents and ease health-related pressures (Spycher et al., 2021). Furthermore, in an environment where autonomy is already severely restricted (Larsen & Drabiak, 2022), people in prison should be involved in discussions about dose reductions or medication discontinuations to empower them to take an active role in managing their health (Avieli, 2023). Moreover, addressing issues such as medication delays, missed doses, and non-adherence through better coordination between nurses, prescribers, and GPs is needed (Bowstead & Meek, 2024), which can also positively impact resource allocation of primary care and mental health services following release (Favril et al., 2024).

There was a thread throughout the analysis about the importance of the relationship between staff and people in prison. Participants identified interpersonal hostilities, wherein prison officers can be viewed as gatekeepers to healthcare access. This presents as appointments (internally and externally) being missed or cancelled due escort unavailability but being attributed to the person in prison. The lack of coordination and communication between teams, but also between staff and people in prison contributes to the deterioration of these relationships. The importance of good relationships between staff and those residing in prison has been recognised as central to the smooth running of prisons (Crewe et al., 2015), so too is the coordination between prison and healthcare staff (Blackaby et al., 2023). This is made more challenging by persistent issues with staffing levels and the difficulties of hiring and retaining staff (both residential and healthcare) (Winship et al., 2019). Dependence on agency staff can impact the consistency of care, especially when staff are unfamiliar with working in the unique prison environment. Previous work on patient safety in prison has recommended sufficient training and clear policies are needed to improve this situation (McFadzean et al., 2023).

Ensuring staff are adequately trained about trauma and mental health will also improve care delivery and relationships between staff and people in prison (Inquest, 2020). To bridge the gap between clinical care and daily supervision, prison staff need clear and accessible plans to better support the needs of people in prison. This would involve exploring how communication between prison departments and healthcare teams can be strengthened to improve coordination, responsive care and individual welfare (Spycher et al., 2021). There is a need to explore what information regarding individuals’ health can be shared with prison staff without compromising confidentiality. For example, a multi-method analysis comparing patient safety incidents in community and prison healthcare identified significant inter-team communication issues, negatively impacting effective medical referrals and information transfer (McFadzean et al., 2023). Moreover, though failure to recognise clinical deterioration was present in both contexts, harm stemmed from inadequate escalation. Therefore, simplifying referral pathways for custodial staff to act more swiftly on health concerns has the potential to prevent deterioration and the requirement for emergency care. Making the appointment system more transparent and accountable could increase trust in the healthcare process and reduce frustration for all. Investing in telemedicine or in-cell phones may reduce the need for staff escorts and broaden access to care, while the development of accessible, visually supported health education materials on key topics would promote better health literacy across the prison population.

### Strengths and limitations

The inclusion of the perspectives of both staff and people in prison is essential in the realm of patient safety and has provided an explanation of specific systemic failures perceived to underpin experiences of avoidable harm. Using a purposive approach to sampling means we have captured views across different types of prison which is important when considering the transferability of the results. However, perspectives were only derived from five prisons and thus, we acknowledge that although transferable, the results do not necessarily represent the broader prison estate and staff and people in prison more generally. There is an argument for exploring the healthcare experiences of women in prison more specifically as well as considering young offenders who have distinct needs and utilise and interact with services differently (Harris et al., 2007). Nevertheless, the current study adds depth to our understanding of avoidable healthcare harm in prison from the perspectives of staff and people in prison.

### Implications for policy and practice

Our analysis suggests multiple mechanisms through which system constraints can translate into harm. Resource limitations and organisational fragmentation (e.g., staffing shortages, poor coordination and communication) can constrain the capacity of prison healthcare to deliver care. These constraints manifest in process failures such as delays, discontinuities, and communication gaps and could increase the likelihood of harm. Some components of the pathways identified by this analysis may extend beyond the study setting discussed here. Features such as escort dependence and restricted autonomy are characteristic of many custodial systems and are therefore likely to generate similar constraints on care delivery across contexts. However, the way in which these constraints translate into practice will depend on the specific setting, for example staffing configurations, service organisation and infrastructure.

A systems-based approach is needed to improve coordination between services; this could include clarifying roles and responsibilities, fostering learning from safety events, and exploring the use of technology and digital solutions to support integration and communication. A recent investigation report exploring prison data sharing and IT from the Health Services Safety Investigations Body (HSSIB) found the IT systems lack interoperability with important information about individual location, healthcare appointments, and clinical or security risks not being reliably shared between systems (Health Services Safety Investigation Body, 2025). The inefficient and oft error-prone manual processes compromise patient safety and the report recommends that both HM Prison and Probation Service and NHS England ensure future prison and healthcare IT systems are fully interoperable to better support the safety and wellbeing of staff and people in prison (Health Services Safety Investigation Body, 2025).

Any interventions to improve patient safety should consider the perspectives of both staff and people in prison to allow for important contextual insights into the prison environment which can help shape services to ensure they are fit for purpose. For example, Edge et al. (2020) offer telemedicine as a cost-effective approach, which has been well received by people in prison and prison staff alike and a systematic review has found this method to be successful in overcoming regime-imposed barriers (Tian et al., 2021). However, its effectiveness requires appropriate trialling to assess the suitability of its use, for example, where people in prison require specialist care. There is also arguably a need for interventions led by people in prison and improved health literacy to increase their autonomy and reduce feelings of hopelessness in relation to their health. This could include exploring better access to exercise, considering how a healthy lifestyle within prison could be fostered and how people could be better supported to attend appointments.

## Conclusions

Our findings illuminate understanding of avoidable healthcare-associated harm in prisons from the perspectives of staff and people in prison. Challenges in preventing avoidable healthcare harm centered on issues with medication management, logistical and staffing challenges and problems inherent within the system in terms of collaboration and communication. The findings suggest several actionable system changes; these include strengthening referral pathways, improving accountability for appointment scheduling, enhancing communication between staff, and supporting greater autonomy in care. These areas map directly onto the challenges described by participants and indicate where system-level changes may be beneficial. However, the hard work of prison custodial and healthcare staff in such challenging environments needs to be acknowledged and whilst it was beyond the scope of this paper, examples of good practice do exist. Considering quantitative data on the scale and severity of avoidable harm in prisons, alongside the voices of staff and people in prison here, will help to guide the reforms needed to improve patient safety (McFadzean et al., in press). Ultimately, it is likely that a multi-layered strategy targeting policy, public perception, professional standards, and prison culture is needed to meet the healthcare needs of people in prison and meet the principle of equivalence.

## Data Availability

No data are available. The participants of this study did not give written consent for their interview transcripts to be shared beyond our research team and when taken in their entirety, may render individuals identifiable.
